# Large-scale production of CdO/Cd(OH)_2_ nanocomposites for non-enzyme sensing and supercapacitor applications†

**DOI:** 10.1039/c7ra09457d

**Published:** 2018-01-03

**Authors:** Mohamed Khairy, Haytham A. Ayoub, Craig E. Banks

**Affiliations:** Chemistry Department, Faculty of Science, Sohag University 82524 Egypt mohamed.khairy@science.sohag.edu.eg +20 01092099116; Faculty of Science and Engineering, Manchester Metropolitan University Chester Street Manchester M1 5GD UK

## Abstract

Recent advancements in electrode design are substantially linked to state-of-the-art nanomaterial fabrications. Herein, we report a simple one-pot hydrothermal synthesis of Cd(OH)_2_ with a platelet-like morphology, which was subsequently annealed at relatively high temperatures to produce a CdO/Cd(OH)_2_ nanocomposite for the first time. It was found that the control of thermal treatment allowed tunable charge transport across the nanometre scale due to the presence of CdO and Cd(OH)_2_ mixed nanocrystals. The CdO/Cd(OH)_2_ nanocrystals offer interesting prospects for the electrocatalytic oxidation of nitrite ions and for supercapacitor applications. The CdO/Cd(OH)_2_ nanocomposite was blended with a trace amount of gold NPs for enhancing the electrochemical conductivity and electrocatalytic capability for nitrite oxidation with a sensitivity of 32.9 μA mM^−1^. It afforded a promising electrocatalyst in a wide concentration range up to 10 mM with a low detection limit of 0.87 μM. Furthermore, the CdO/Cd(OH)_2_ nanocomposite electrode was showed to be a highly active and stable supercapacitor, achieving a high specific capacitance in an alkaline medium of about 145 F g^−1^ at a discharge current of 2.0 A g^−1^. These results have revealed that the presence of mixed oxide/hydroxide nanocrystals in nanoscale dimensions will be very interesting for various electrochemical applications and provide for a new class of nanodevices based on electrochemistry with unique capabilities.

## Abstract 2

A simple fabrication of CdO/Cd(OH)_2_ nanocomposites was developed and explored for electrochemical-based devices. The nanocomposite is shown to be a sensitive electrode material for nitrite determination in water samples as well as a promising supercapacitor.

## Graphical abstract

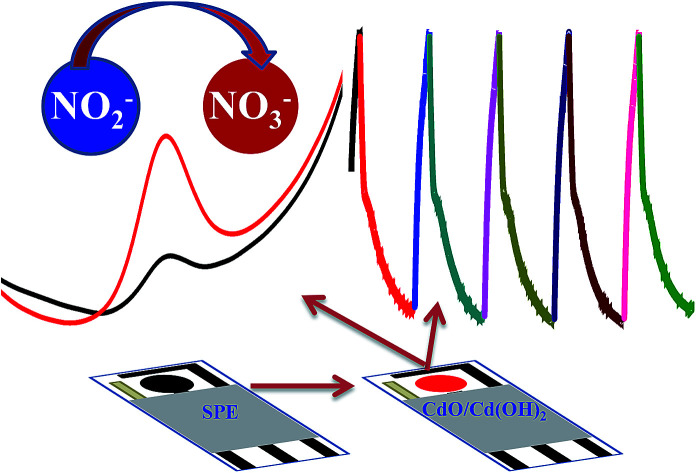

## 1. Introduction

Transition metal oxide/hydroxide nanostructures are widely applied in a plethora of technologically significant disciplines such as optoelectronic,^1^ catalysis,^2^ dye-sensitizer solar cell,^3^ and sensor applications.^4,5^ Because they provide a high surface area to volume ratio, hydrothermal stability, and various oxidation states, they possess unique optical, electronic, and magnetic properties.^6,7^ Interestingly, the morphological-dependent interatomic bond distances, stoichiometry, and crystal defects have profound influences on their functionality and applicability.^8^ Therefore, considerable efforts have been exerted to control the intrinsic crystal arrangement and morphology of the nanomaterials to enable specific functionalities that are selected to align with the targeted applications. Among the various transition metal hydroxides, Cd(OH)_2_ nanostructures have gained significant interest because they can be potentially converted to CdO through dehydration or to other Cd-based functional materials (*e.g.* CdO, CdS, and CdSe) by appropriate reaction with other elements or compounds.^9^ Cd(OH)_2_ is a highly promising material for optoelectronic and other applications such as in solar cells,^10,11^ phototransistors,^12^ photodiodes,^13^ transparent electrodes, cathode electrode of batteries, and gas sensors.^14^ Various morphologies of Cd(OH)_2_, such as nanoplatelets,^9^ nanorings^15^ and nanowires,^16^ have been reported recently. Despite this significant progress, it is still a big challenge to align Cd-based nanostructures for various targeted applications.

Recently, the areas of electrochemistry (*i.e.* electrocatalysis, supercapacitors, batteries, fuel cells, solar cells and electroanalytical chemistry) have been greatly impacted by the availability of materials in nanoscale dimensions in term of their size, morphology, porosity and functionality, because they offer a short diffusion path and a favourable electrode/electrolyte contact area. These significant benefits can lead to the potential development of novel nanodevices with unique functionalities and processes. Therefore, the development of nanostructured electrodes is a very active and robust research area that is expected to provide next-generation technologies for environmental and food science analyses, improving and/or supplanting conventional sensing strategies. Although, nitrites are toxic inorganic contaminants, they have been widely used in meat curing,^17^ food preservatives,^18^ dyes, bleaches, fertilizers and in many medicinal purposes.^19^ The nitrites in water and the atmosphere are essential precursors in the formation of carcinogenic nitrosamines in the stomach.^20^ Significantly, nitrites can oxidize haemoglobin to methaemoglobin, which is not capable of carrying oxygen; furthermore, the latter can reach toxic concentrations in a high density aquaculture system and as a result, the higher concentration of nitrites leads to considerable economic losses to aquaculture production.^21^ The fatal dose of nitrite ingestion is reported to be between 8.7 μM and 28.3 μM and consequently the World Health Organization has mentioned that nitrites should have a guidance level of 3 mg L^−1^ (65 μM) for short-term exposure and a provisional guideline level of 0.2 mg L^−1^ (4.3 μM) for long-term exposure.^19^ Therefore, the development of reliable, portable, sensitive and economical sensors for monitoring nitrites is highly recommended.^22^

To realize the objective of nanostructured electrodes, different transition metal oxides nanostructures, such as RuO_2_, Fe_2_O_3_, Co_3_O_4_, NiO, MnO_2_, CuO, have been reported for high-power electrochemical supercapacitors.^23,24^ Although the toxicity of cadmium-based materials is well known, Cd(OH)_2_ has been widely used as an electrode material for cadmium–nickel batteries because of its high energy density, long lifetime, high discharge rates and relatively low cost. Cd(OH)_2_ nanowires have been recently explored as low-cost alternative electrode materials for supercapacitors. Cd(OH)_2_ nanowires synthesized by a chemical bath deposition method on a stainless-steel substrate yielded a specific capacitance of 46.15 F g^−1^ at 0.93 A g^−1^. Thus, simplicity, flexibility and efficient power performance are still the major challenges for the development of Cd(OH)_2_ and/or CdO supercapacitors. In the present work, we built an advanced nanostructured electrode based on a CdO/Cd(OH)_2_ nanocomposite with a platelet-like morphology. First, Cd(OH)_2_ nanoplatelets were synthesized *via* a simple and scalable hydrothermal protocol. Then, a subsequent low-temperature annealing process was performed to reveal CdO/Cd(OH)_2_ mixed nanocrystals. To optimize the electrode performance and minimize the resistivity, a trace amount of citrate-capped gold nanoparticles was added to the CdO/Cd(OH)_2_ nanoplatelets dispersion. The nanocomposite suspension was then drop-cast on the surface of screen-printed electrodes (SPEs). Interestingly, the CdO/Cd(OH)_2_ nanocomposite-modified SPEs represented mechanically robust, stable, portable, flexible, low-cost and low-resistive nanostructured electrodes that exhibited promising electrocatalytic activity towards nitrite oxidation and for supercapacitor applications.

## 2. Experimental

### 2.1. Chemicals

All the chemicals were of the highest analytical grade available and were used as received without further purification. Cadmium chloride (CdCl_2_), ammonium hydroxide (NH_4_OH 28%), ethanol, disodium hydrogen phosphate (Na_2_HPO_4_), potassium chloride (KCl), sodium sulfate (Na_2_SO_4_·6H_2_O), sodium nitrite (NaNO_2_), sodium nitrate (NaNO_3_), sodium iodide (NaI), sodium thiosulfate (Na_2_S_2_O_3_), sodium acetate (CH_3_COONa), boric acid and ascorbic acid were supplied from Sigma-Aldrich Co. Ltd. All the solutions were prepared using bi-distilled water with a resistivity of more than 18.2 MΩ cm. A 0.05 M Britton–Robinson buffer solution was prepared by mixing equimolar amounts of boric acid, citric acid and phosphoric acid solutions. The pH was adjusted by the addition of 1.0 M sodium hydroxide solution.

### 2.2. Synthesis of CdO nanostructures

Cd(OH)_2_ nanoplatelets were fabricated *via* a hydrothermal route. First, 1.83 g of CdCl_2_ was dissolved in 50 mL water and a measurable volume of NH_4_OH solution (28%) was added dropwise under magnetic stirring to adjust the pH to 9.5. Subsequently, the solution was transferred into a 100 mL Teflon autoclave and heated at 160 °C for 6.0 h. The obtained Cd(OH)_2_ was washed several times with a mixture of deionized water and ethanol to remove the remaining agents and then dried at 60 °C overnight. To obtain CdO/Cd(OH)_2_ and CdO nanoplatelets, a subsequent annealing process was performed in air at 300 °C and 400 °C, respectively, for 3.0 h with a heating rate 2 °C per minute (Fig. S1 in ESI†).

### 2.3. Fabrication of the screen-printed electrodes

The SPEs were fabricated in-house with an appropriate stencil using a DEK 248 screen-printing machine (DEK, Weymouth, UK). These electrodes have been used extensively in previous studies.^5,8,25–27^ Here, first, a carbon-graphite ink formulation (product code C2000802P2; Gwent Electronic Materials Ltd, UK) was screen-printed onto a polyester (Autostat, 250 μm thickness) flexible film (denoted throughout as standard-SPE). This layer was cured in a fan oven at 60 °C for 30 min. Next, a silver/silver chloride reference electrode was included by screen-printing Ag/AgCl paste (product code C2040308D2; Gwent Electronic Materials Ltd, UK) onto the polyester substrates and a second curing step was undertaken where the electrodes were cured at 60 °C for 30 min. Finally, a dielectric paste (product code D2070423D5; Gwent Electronic Materials Ltd, UK) was then printed onto the polyester substrate to cover the connections. After a final curing at 60 °C for 30 min, these SPEs were ready to be used. These SPEs have been reported previously and shown to exhibit a heterogeneous electron transfer (HET) rate constant, *k*^o^, of *ca.* 10^−3^ cm s^−1^, as measured using the [Ru(NH_3_)_6_]^3+/2+^ redox probe.

### 2.4. Fabrication of CdO/Cd(OH)_2_-nanocomposite-modified screen-printed electrodes

The SPE was modified with CdO/Cd(OH)_2_ nanocomposite by a simple drop-cast process. A stock solution of CdO/Cd(OH)_2_ nanocomposite was prepared by dissolving 10.0 mg of the CdO/Cd(OH)_2_ sample in 10.0 mL double distilled water and then 1.0 mL (0.3 mg) of citrate-capped gold nanoparticles was added and mixed well. The nanocomposite suspension was stored at 4 °C.^28^ A measured volume of CdO/Cd(OH)_2_/Au suspension was dropped onto the SPE surface and left to dry for 30 min in a desiccator. Then, the CdO-nanocomposite-modified screen-printed electrode was ready to use for further electrochemical experiments.

### 2.5. Water sample analysis

A sample of tap water was collected and its pH was adjusted by the addition of sulfuric acid solution (0.1 M). Then, differential pulse voltammetry (DPV) was performed on the solution and the responses recorded, while a standard addition method was utilized to determine the concentration of nitrite ions in the tap water.

### 2.6. Characterizations of the CdO/Cd(OH)_2_ nanostructures

The morphology of Cd(OH)_2_ samples before and after thermal treatment were investigated using field emission scanning electron microscopy (FE-SEM, JEOL model 6500). Before inserting the sample into the chamber, the powder was ground and fixed onto a specimen stub using double-sided carbon tape. Then, a 10 nm Pt film was coated *via* anion sputtering (Hitachi E-1030) at room temperature to obtain high-resolution micrographs. For better recording of the FE-SEM images, the SEM was operated at 15 keV.

Wide-angle powder X-ray diffraction (XRD) patterns were measured using an X-ray diffractometer (model FW 1700 series, Philips, Netherlands) with monochromated CuKα radiation (*λ* = 1.54 Å), employing a scanning rate of 0.060 min^−1^ and 2*θ* ranges from 4° to 60°. The diffraction data were analysed using the DIFRAC plus Evaluation Package (EVA) software with the PDF-2 Release 2009.

The textural surface properties and pore-size distribution were determined by N_2_ adsorption/desorption isotherms at 77 K with a BELSORP analyser (BEL Co., JP Ltd). The specific surface area (*S*_BET_) was calculated using the Brunauer–Emmett–Teller (BET) method with multipoint adsorption data from the linear segment of the N_2_ adsorption isotherm. The pore-size distribution was determined from analysis of the desorption branch of the isotherm using the nonlocal density functional theory (NLDFT). Furthermore, thermogravimetric analysis (TGA) was measured in a flow of nitrogen using TG-60 (Shimadzu Ltd, JP).

The electrochemical experiments were performed using an Autolab 302 N potentiostat/galvanostat workstation. All the measurements were conducted using a screen-printed electrode configuration. During the development of the protocol, the CdO/Cd(OH)_2_-nanocomposite-modified screen-printed electrode was used with a platinum counter electrode and Ag/AgCl/KCl_(sat)_ as the reference electrode. Connectors for the efficient connection of the screen-printed electrochemical sensors were purchased from Kanichi Research Services Ltd (UK).^25–28^

## 3. Results and discussion

### 3.1. Features of the CdO/Cd(OH)_2_ nanocomposites

**Fig. 1:**
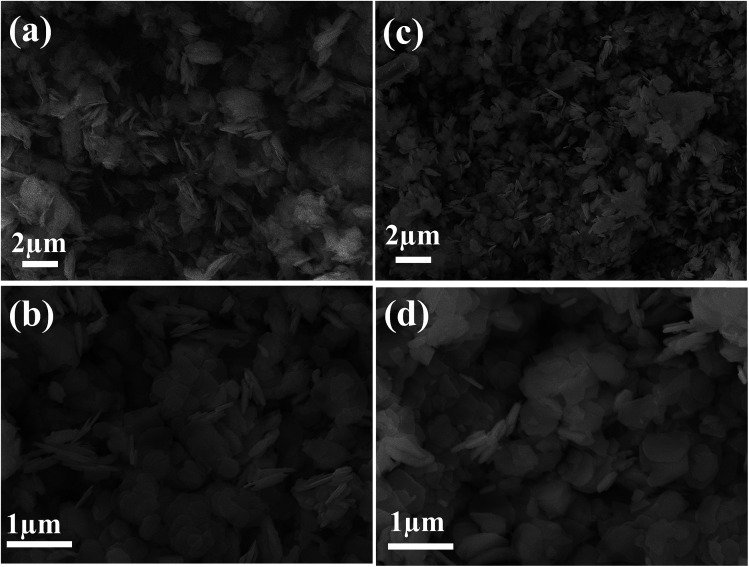
SEM images of Cd(OH)_2_ samples before (a and b) and after (c and d) treatment at 300 °C.

The formation of the CdO/Cd(OH)_2_ nanoplatelets *via* a template-free mechanism mainly involved nucleation, growth and dehydroxylation steps. First, the addition of ammonium hydroxide to CdCl_2_ solution generated small nuclei Cd(OH)_2_, which then subsequently dissolved in the presence of excess ammonia solution due to the formation of [Cd(NH_3_)_*n*_]^2+^ ions. Under hydrothermal conditions, the solubility of the [Cd(NH_3_)_*n*_]^2+^ complex is decreased due to the formation of NH_3_ gas bubbles. These gas bubbles can be used as a template to guide developing small nuclei Cd(OH)_2_ (*k*_sp_ = 7.2 × 10^−15^) and to grow Cd(OH)_2_ with a hexagonal plate-like shape by an Ostwald ripening process. Finally, the dehydroxylation of as-prepared Cd(OH)_2_ under mild thermal treatment at 300 °C led to formation of CdO/Cd(OH)_2_ mixed nanocrystals with a platelet-like morphology. However, CaO nanoplatelets were produced after thermal treatment at 400 °C, as shown in the TG analysis (Fig. S1†). Furthermore, it was noted that no precipitate was formed without the addition of ammonium hydroxide solution. When replacing NH_4_OH by NaOH solution, Cd(OH)_2_ plates with an irregular shape were obtained. When increasing the solution pH to 12.0, no change in morphology or in the size of the plate was observed (Fig. S2†). Fig. 1 shows the scanning electron microscopy (SEM) images of the as-synthesised Cd(OH)_2_ sample before and after thermal treatment at 300 °C. The Cd(OH)_2_ had a hexagonal platelet-like morphology, which was maintained even after thermal treatment. It was found that the dehydroxylation of Cd(OH)_2_ at 300 °C did not change the morphology or size. The as-synthesised Cd(OH)_2_ and CdO/Cd(OH)_2_ particles had nano-sized dimensions and smooth fine surfaces with an average diameter of 200–300 nm and a thickness of about 30 nm. The SEM micrographs showed that the simple synthesis procedures could efficiently control the shape and size of Cd(OH)_2_ and CdO in condensed orientation sequences (as shown in Fig. S2†).

**Fig. 2:**
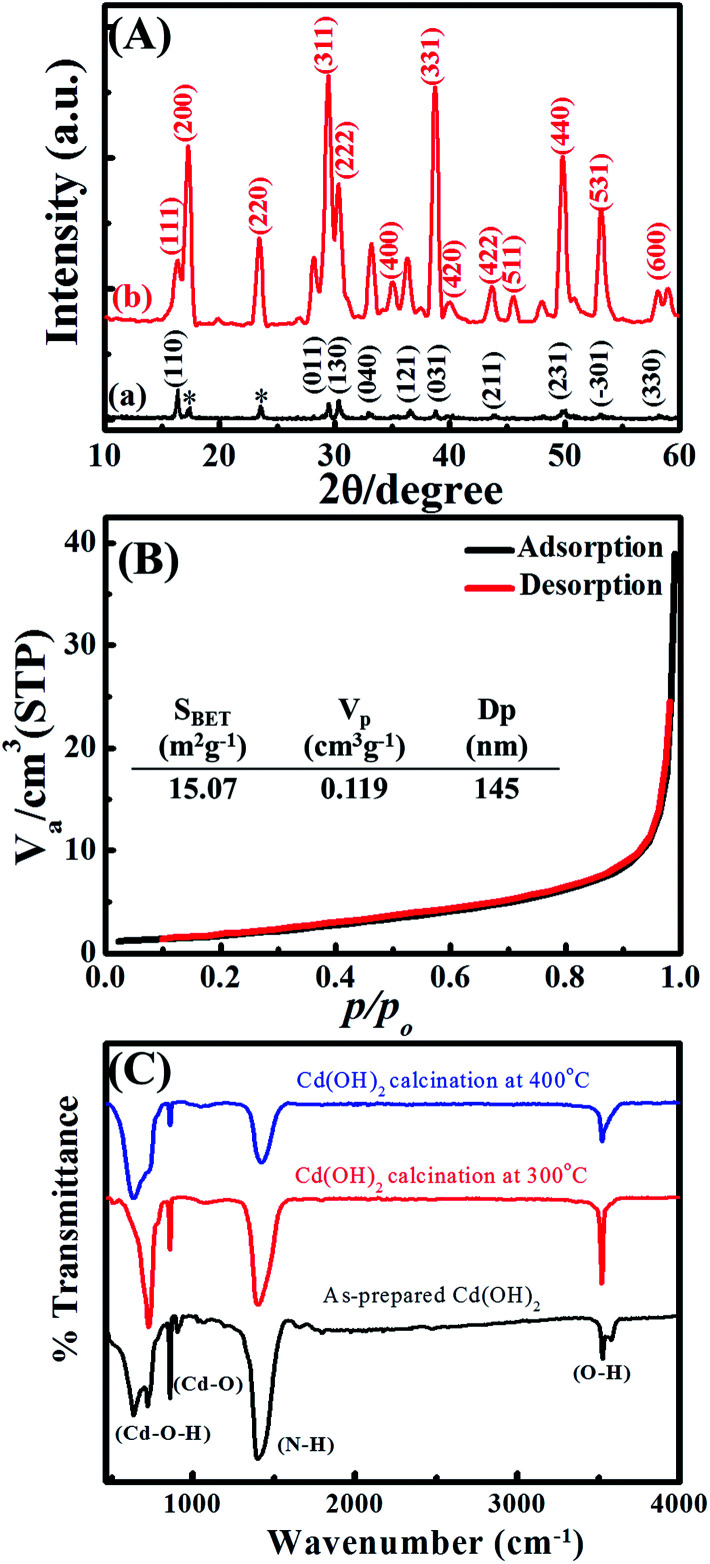
(A) Wide-angle X-ray diffraction, (B) N_2_ adsorption/desorption isotherms and (C) FTIR spectra of Cd-based materials.

To identify the crystal structure of cadmium-based materials before and after thermal treatment, wide-angle X-ray diffraction (XRD) was performed. Fig. 2 shows the XRD patterns of the as-prepared Cd(OH)_2_ and thermally treated Cd(OH)_2_ sample at 300 °C, where the well-resolved and distinct diffraction peaks of CdO were assigned to the (111), (200), (220), (311), (222), (400), (331), (420), (422), (511), (440), (531) and (600) planes in addition to the Cd(OH)_2_ diffraction peaks, indicating the presence of CdO/Cd(OH)_2_ mixed nanocrystals in a platelet morphology. The diffraction peaks of the CdO crystals coincided with the cubic (*Fm*3*m*) symmetry of JCPDS card no. 01-1049 and a lattice constant of *a* = 4.689 Å. The typical diffraction peaks of Cd(OH)_2_ crystals assigned to the (110), (011), (130), (040), (121), (031), (211), (231), (−301) and (330) planes corresponded to the monoclinic symmetry of JCPDS card no. 84-1767. The peaks marked with an asterisk (*) were possibly related to ammonium cadmium hydroxide (JCPDS card no. 18-0081). This result indicated that the monoclinic nanocrystals of Cd(OH)_2_ were converted to the cubic (*Fm*3*m*) symmetry of CdO nanocrystals after thermal annealing at a relatively high temperature, which agreed with the conversion of nonconductive monoclinic crystals of La_2_Mo_2_O_9_ to a highly conductive cubic form after thermal treatment at 580 °C.^29^ Thus, the produced CdO/Cd(OH)_2_ nanoplatelets composite *via* the dehydroxylation of Cd(OH)_2_ at 300 °C might be very interesting for various electrochemical applications.

The textural properties of the CdO/Cd(OH)_2_ nanoplatelets were investigated by N_2_ adsorption/desorption isotherms (Fig. 2B). The CdO/Cd(OH)_2_ nanocomposite showed a type II isotherm without any hysteresis loops, indicating the formation of macropore cavities between the nanoplatelets. The CdO/Cd(OH)_2_ nanocomposite exhibited a low specific surface area of *S*_BET_ = 15.07 m^2^ g^−1^ with a macropore distribution of 145 nm. The porous network texture of CdO/Cd(OH)_2_ is very important for designing electrochemical-based devices because it provides an enhanced surface area and alters the mass transport characteristics.

To determine the surface functional groups of Cd-based materials before and after thermal treatments, FTIR spectra were collected, with the results shown in Fig. 2C. The CdO/Cd(OH)_2_ nanoparticles show absorption bands below 1000 cm^−1^, corresponding to Cd–O and Cd–O–H stretching vibrations, which confirm the formation of CdO and CdO/Cd(OH)_2_ nanoparticles after thermal treatment at 400 °C and 300 °C, respectively. Whereas, the band at 3530 cm^−1^ is attributed to the stretching vibration of the hydroxyl group, indicating the presence of residual water and hydroxyl groups in the CdO nanoparticles. The absorption bands at 1400 cm^−1^ can be attributed to N–H stretching. The FTIR investigations clarified that the cadmium oxide particles involve a trace amount of Cd(OH)_2_ nanocrystals as well as containing N–H bond intercalates within the oxide phase.^16^ With increasing the thermal temperature, the absorption bands at 3526, 1400 and 856 cm^−1^ were significantly decreased due to the formation of CdO nanocrystals.

### 3.2. Electrochemical sensing of nitrites on CdO/Cd(OH)_2_ nanocomposites

#### 3.2.1. Voltammetric behaviour of nitrite ions

**Fig. 3:**
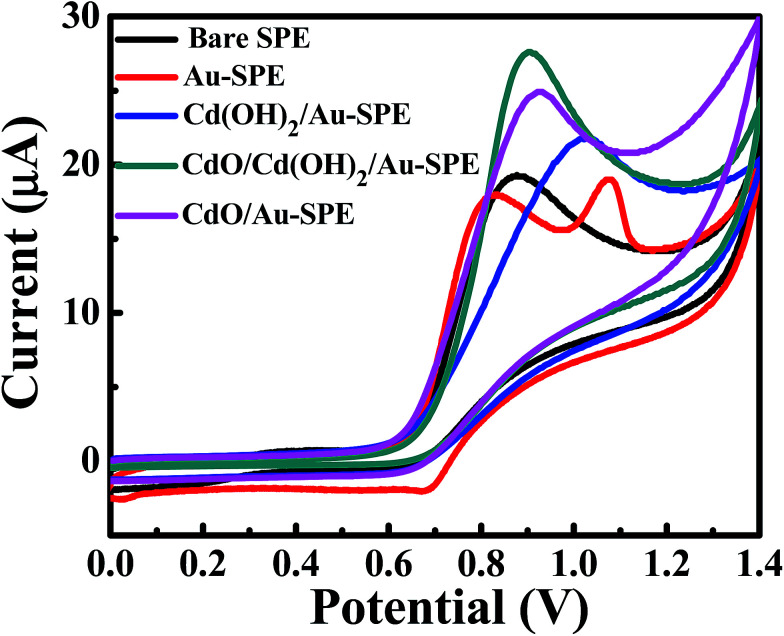
CV curves of 1.0 mM sodium nitrite in 0.1 M Na_2_SO_4_ (pH 4) on unmodified/bare SPEs: Au-SPE, Cd(OH)_2_/Au-SPE, Cd(OH)_2_/CdO/Au-SPE and CdO/Au-SPE at a scan rate of 0.05 V s^−1^.

Unfortunately, the electrochemical oxidation of nitrites on the bare electrodes caused accumulative electrode-passivation/fouling effects, which greatly limited the analytical performance of the electrodes.^30^ Thus, chemically modified electrodes have attracted great attention in the last decades. Fig. 3 shows the cyclic voltammetry (CV) responses of 1.0 mM sodium nitrite in 0.1 M sodium sulfate Na_2_SO_4_ at pH 4 on unmodified/bare SPEs, Au-SPEs, Cd(OH)_2_/Au-SPEs, CdO/Cd(OH)_2_/Au-SPEs and CdO/Au-SPEs at a scan rate of 0.05 V s^−1^. No noticeable reduction peak can be observed, indicating that the electro-oxidation of nitrite ions on the cadmium oxide composite-modified screen-printed electrode is irreversible in nature. Although citrate-capped gold nanoparticle shift the oxidation peak of nitrites to a more negative potential, the peak current does not change compared to bare SPEs, and a new oxidation peak appears, which was attributed to the oxidation of gold nanoparticles assembled on the SPEs. The Cd(OH)_2_/Au-SPEs, CdO/Cd(OH)_2_/Au-SPE and CdO/Au-SPE showed enhanced peak current for nitrite oxidation with a positive potential shift of about 140, 10 and 40 mV *vs.* Ag/AgCl, respectively. The positive potential shift of Cd(OH)_2_ was higher than for the CdO material, despite them having almost the same size and platelet-like morphology. Thus, the cubic crystal CdO nanocrystals have a higher electrocatalytic activity than monoclinic Cd(OH)_2_ nanocrystals due to the conductivity–structure relationship *via* a disorder in the oxygen sublattice.^29^ Furthermore, the presence of Cd(OH)_2_ nanocrystals in the CdO nanoplatelets is very important to enhance the electrochemical performance and electrocatalytic activity of cadmium oxide nanoplatelets.

To find out the best electrochemical response of the CdO/Cd(OH)_2_ nanocomposite for the electrochemical determination of nitrites, the effects of the supporting electrolytes, the solution pH and the mass loading of composite material on the SPEs were explored. Fig. S3† shows the cyclic voltammetry response of 0.5 mM nitrite on CdO nanocomposite-SPE in the presence of 0.1 M various supporting electrolytes, namely sodium sulfate, sodium hydrogen phosphate and potassium chloride, at a scan rate 50 mV s^−1^. It was found that the anodic peak current of nitrite was enhanced in the presence of 0.1 M sodium sulfate as a supporting electrolyte (Fig. S4†). Furthermore, the voltammetric response towards the electrochemical oxidation of nitrite was also investigated on the CdO nanocomposite-SPE in nitrite solutions of differing pHs (Fig. S5†). The pH of 0.1 M sodium sulfate was adjusted by adding a few drops of sulfuric acid and sodium hydroxide to reach the required pH value. Analysis of the voltammetric peak current, *I*_p_, as a function of pH is described in Fig. S5b.† It was found that the voltammetric peak current was increased up to pH 4 due to the instability and protonation of nitrite ions at a very low pH. Meanwhile, the peak current does not change at higher pH values, indicating that the electrocatalytic oxidation of nitrite is proton independent. Thus, pH 4 was selected as a suitable pH value to determine nitrite ions in water samples. Furthermore, the voltammetric peak potential, *E*_p_, was shifted to less positive values with increasing the pH of the nitrite solution. This result revealed the participation of protons in the electrochemical oxidation process of nitrites. Beyond pH 4, the peak potential was insensitive to pH, which is consistent with the reported p*K*_a_ for nitrous acid between 3.2 and 3.4. Also observed was a linear response of the peak potential from the oxidation of nitrite at pH lower than 4 with a slope of 55 mV per pH. This value is very close to the Nernstian slope (59 mV per pH at 298 K) for a process that involves the transfer of an equal number of protons and electrons in an electrochemical process. The mass loading of CdO nanocomposites onto SPEs was also studied (Fig. S6†). It was obvious that 5.0 μg of CdO nanocomposites on SPE was sufficient for the electrocatalytic oxidation of nitrite solution. Thus, a 0.1 M Na_2_SO_4_ solution at pH 4 and 5.0 μg of CdO nanocomposite-SPEs were used in the further voltammetric experiments of the nitrite sensing.

**Fig. 4:**
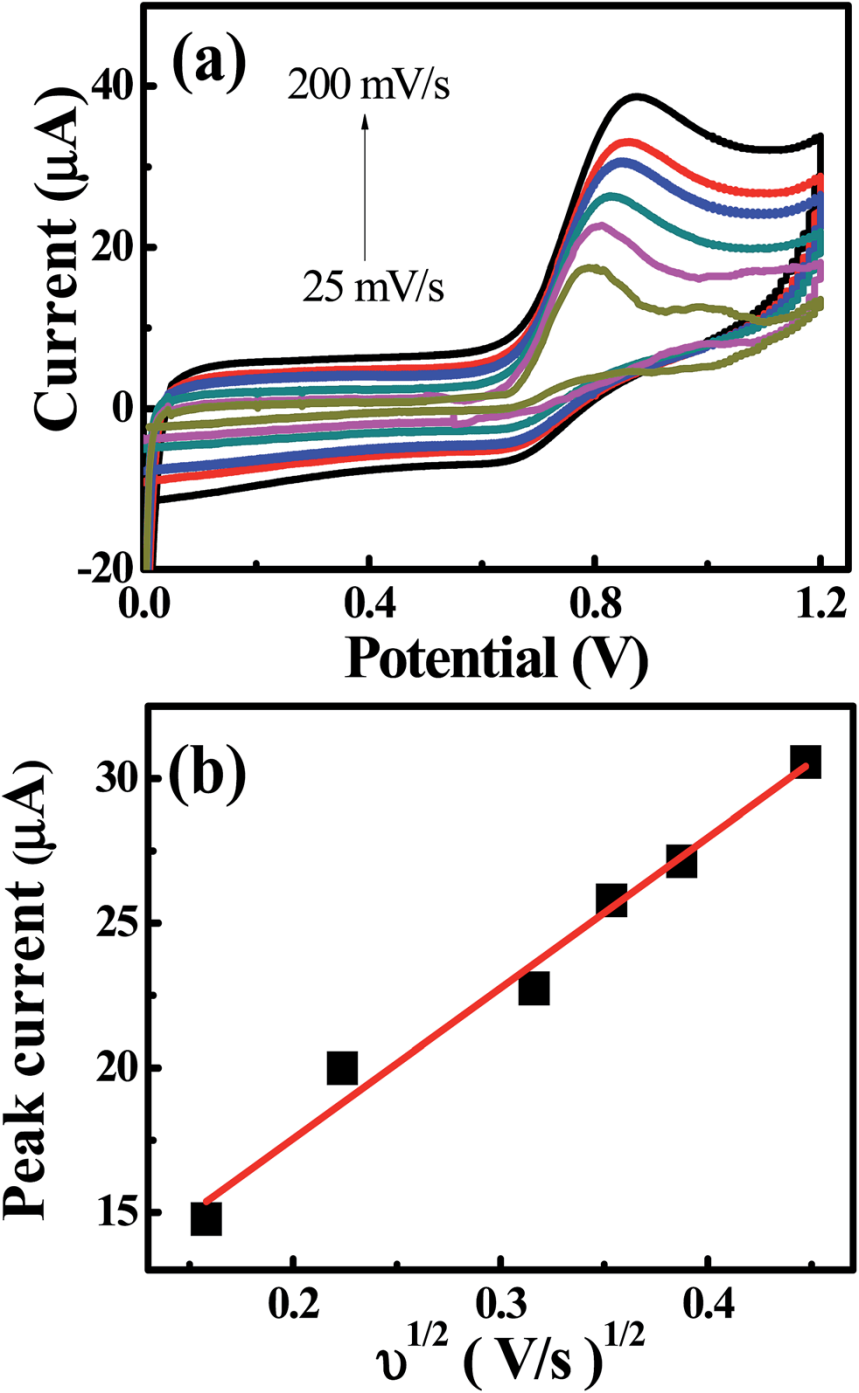
(a) Typical CV curves of 1.0 mM sodium nitrite in 0.1 M Na_2_SO_4_ (pH 4) and (b) dependence of the peak currents on the square root of the scan rate on CdO nanocomposite-modified SPEs.

The cyclic voltammetric experiments were performed at pH 4 over a range of scan rates from 25 to 200 mV s^−1^, as shown in Fig. 4. The voltammetric current signal-scan rate dependence for all scan rates was observed; whereby, upon increasing the scan rate, the current magnitude increases, where the peak current is proportional linearly to the square root of the scan rate; *I*_p_/μA = 52.0*ν*^1/2^/*V*^1/2^*s*^−1/2^ + 7.13 μA. This result suggested that the oxidation of nitrite on the CdO nanocomposite-SPEs is controlled by a mass transfer process. The linear relation of the logarithm of the anodic peak current and the logarithm of the scan rate revealed a slope of 0.4, which is close to 0.5, indicating that there was no adsorption of nitrite molecules on the CdO nanocomposite-SPEs (Fig. S7a†). That there was no reverse reduction wave of nitrite and the positive shift in the oxidation peak potential confirmed the irreversible nature of the electrode processes. For a totally irreversible diffusion-controlled process, the analysis of the peak potential against the scan rate showed a linear segment (Fig. S7b†) with a slope of 0.095. By applying the following equation:
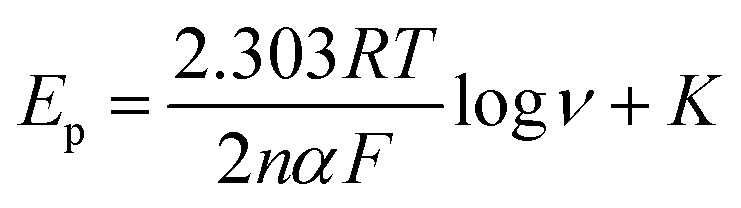
where *E*_p_ is the peak potential, *ν* is the scan rate, *K* is a constant, *α* is the charge transfer coefficient, *R* is a gas constant, *T* is the temperature – 298 K, *F* is the Faraday constant and *n* is the number of electrons consumed in the electrochemical reaction. For a one electron transfer process, the electron transfer coefficient was 0.68, indicating that the oxidation of nitrite on the CdO composite electrode is limited by a mass transfer process. Furthermore, the decrease in the current function (*I*/*ν*^1/2^) with increasing the scan rate revealed that the electrochemical oxidation mechanism of nitrite on the CdO-composite-modified SPE is likely to be *via* an EC mechanism, as follows:NO_2_^−^ − e ⇔ NO_2_2NO_2_ + H_2_O → NO_3_^−^ + NO_2_^−^ + 2H^+^

#### 3.2.2. Electroanalytical sensing of nitrite ions

**Fig. 5:**
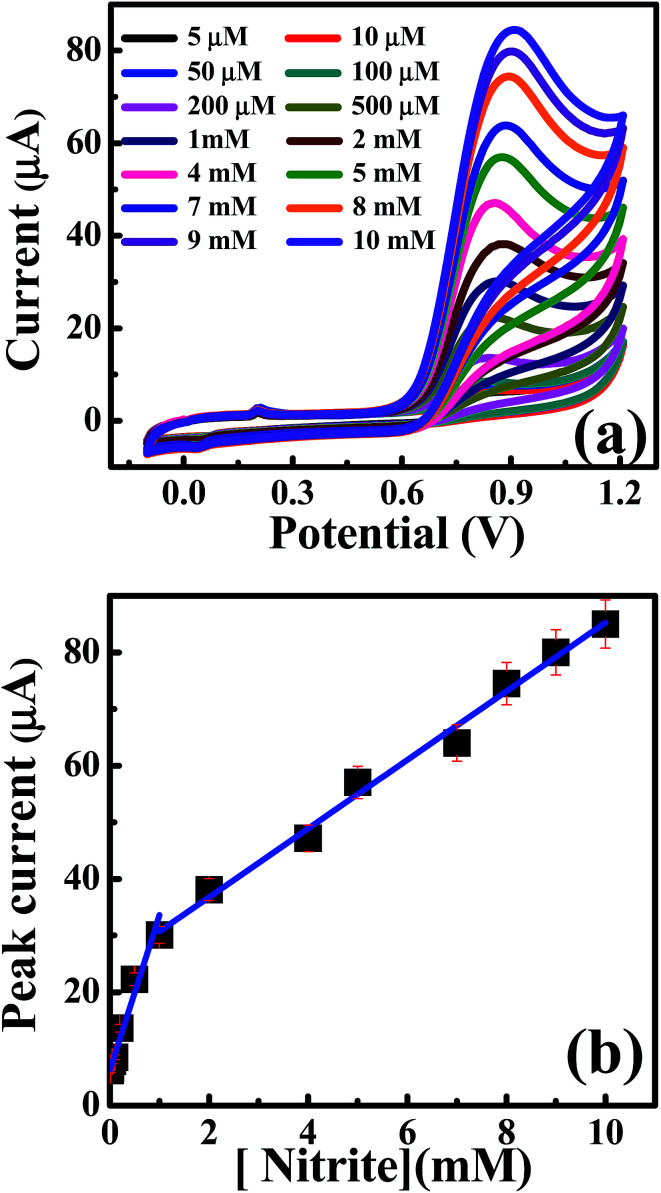
(a) CV curves of nitrite additions in 0.1 M Na_2_SO_4_ at a scan rate of 50 mV s^−1^ on CdO nanocomposite. (b) Analysis of the peak height as a function of the nitrite concentrations.

Next, attention was turned to exploring the electroanalytical performance of the CdO nanocomposite-SPEs towards nitrite detection. Fig. 5a shows the cyclic voltammetric curves resulting from the additions of nitrite concentrations over the range 5.0 μM to 10 mM into the supporting electrolyte of 0.1 M Na_2_SO_4_ at pH 4 at a scan rate of 50 mV s^−1^. A significant enhancement of the oxidation peak current upon nitrite additions was observed, where linear responses were shown over the range of 5.0 μM to 1.0 mM: *I*_p_/μA = 32.86*C*_nitrite_/mM + 6.07 μA; *R*^2^ = 0.99, and 1.0 mM to 10.0 mM: *I*_p_/μA = 6.06*C*_nitrite_/mM + 24.64 μA; *R*^2^ = 0.991. Based on the first linear range, the limit of detection (3S/N) was found to be 0.87 μM.

The selectivity of CdO nanocomposite-SPE towards nitrite determination was evaluated in the presence of some potential interferants, such as sodium acetate, ascorbic acid, boric acid, potassium chloride, potassium iodide, sodium nitrate and sodium phosphate. The CV curves were recorded in solution at pH 4 in the presence of 100 μM nitrite and 100 μM of each interferant. The voltammetric peak height of the nitrite was changed by only about ±3.6% of its original value. Furthermore, the relative standard deviation was 1.18% for five repetitive measurements of 100 μM nitrite ions. Five separately CdO nanocomposite-SPEs were also measured in the presence of 100 μM nitrite ions, where the relative standard deviation was about 3.13%. These results indicated that the CdO nanocomposite-SPE exhibited a high electrocatalytic capability and stability towards the oxidation of nitrites even in the presence of various interferants.

**Fig. 6:**
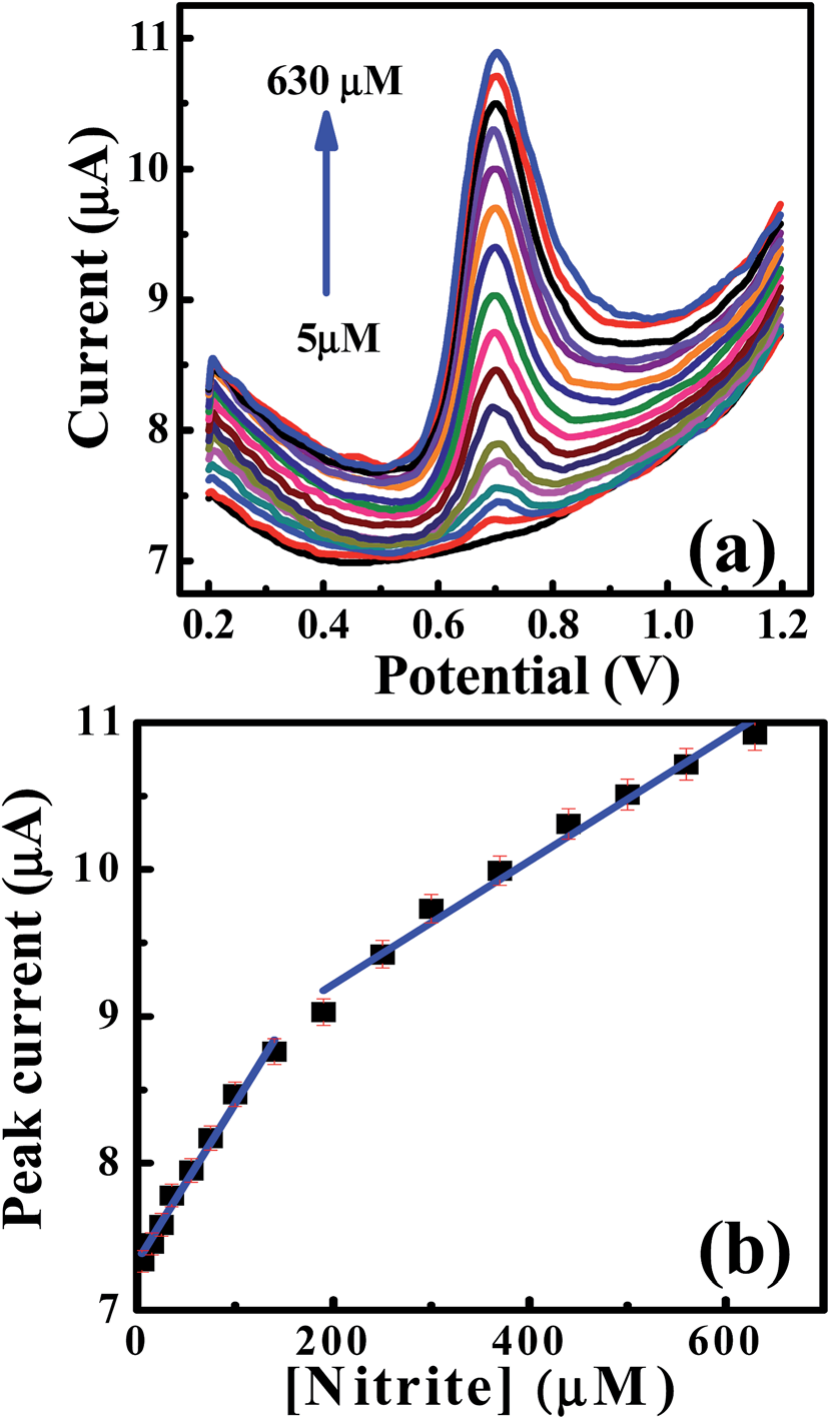
(a) DPV curves for the additions of nitrite into 0.1 M Na_2_SO_4_ on a CdO nanocomposite. (b) Dependence of the peak height on the concentration of nitrite [DPV parameters; step potential = 0.005 V, modulation time = 0.5 s, modulation amplitude = 0.025 V, interval time = 0.05 s and scan rate = 0.05 V s^−1^].

Furthermore, the differential pulse voltammetry (DPV) responses were also explored to increase the sensitivity of the analytical performance of the CdO nanocomposite-SPEs towards nitrite determination. Fig. 6 shows the typical DPV responses for successive additions of nitrites over the range of 5.0–630 μM in 0.1 M of sodium sulfate at pH 4 on the CdO nanocomposite-SPE. Along with increases in the nitrite concentrations, the voltammetric peak currents were linearly enhanced over the range 5.0–150.0 μM: *I*_p_/μA = 0.011*C*_nitrite_/μM + 7.3 μA; *R*^2^ = 0.985, and for 200–630 μM: *I*_p_/μA = 0.004*C*_nitrite_/μM + 8.37 μA; *R*^2^ = 0.98. The limit of detection (3S/N) was deduced to be 0.8 μM, according to the first linear range. Such analytical performance of CdO nanocomposite-SPEs enable the determination of nitrite ions in a wide concentration range with a low detection limit compared to the previously reported literature (Table 1). This could be suitable for short-term exposure detection as described by the World Health Organisation (WHO).^19^ Furthermore, CdO nanocomposite-SPEs offer interesting features, such as low cost, easy construction and storage, a potential for miniaturization and a facility of automation and the construction of simple and portable equipment.

**Table 1:** Comparison of the analytical performance of the proposed CdO composite-modified screen-printed electrode for the determination of nitrite ions with other published work^a^

Electrode	Linear range (μM)	LOD (μM)	Technique	pH	Reference
GR-MWCNTs/FeNPs	0.1–1680	0.076 ± 1.3	Amperometry	5.0	32
HAC-GCE	3.0–90	0.13	DPV	4.0	33
Poly(PyY)/PGE	1.0–100	0.5	Amperometry	4.0	34
Ag-NEEs	10.0–80		CV	7.0	35
GC-electrode	2.5–10	0.4	Amperometry	3.0	36
Pd/RGO/GCE	1–1000	0.23	DPV	7.0	37
Nano-Au/P3MT/GCE	10–1000	2.3	Amperometry	4.0	38
Au-HNT-GO	0.1–6330	0.03	Amperometry	6.0	39
CuO–2TiO_2_	10–200	0.017	LSV	7.2	40
MnO_2_/GO	0.1–1000	0.09	DPV	7.4	41
Cd(OH)_2_/CdO/Au-SPE	5.0–600	0.8	DPV	4.0	Present work
Cd(OH)_2_/CdO/Au-SPE	5.0–10 000	0.87	CV	4.0	Present work

a GR-MWCNTs/FeNPs = iron nanoparticles decorated graphene-multiwalled carbon nanotubes nanocomposite, HAC-GCE = heteroatom-enriched activated carbon modified glassy carbon electrode, Poly(PyY)/PGE = poly pyronin Y on pencil graphite electrode, Ag-NEEs = silver nanoelectrode ensembles, Pd/RGO/GCE = palladium nanoparticles-reduced graphene oxide composite-modified glassy carbon, Nano-Au/P3MT/GCE = gold nanoparticles on poly(3-methylthiophene)-modified glassy carbon electrode, Au-HNT-GO = gold nanoparticles dispersed on halloysite nanotubes-reduced graphene oxide nanosheets, MnO_2_/GO = MnO_2_ decorated graphene oxide.

#### 3.2.3. Detection of nitrites in a tap water sample

To verify the potential application of the CdO composite-modified screen-printed electrode, it was applied for the determination of nitrite in a tap water sample. The DPV responses were explored in a tap water sample after adjusting the pH of the water to 4.0 by adding drops of 0.1 M sulfuric acid. It was found that no voltammetric peak was observed because the concentration of nitrite was below the detection limit capabilities. Consequently, a recovery experiment was performed on the tap water sample. The sample was spiked with different concentrations of nitrites in the range of 20 to 400 μM with a standard addition protocol performed in triplicate (Fig. S8†). It was observed that a recovery of 90 (±3)% was possible, indicating that the CdO composite-modified screen-printed electrode is a promising electrode material for the determination of nitrite in water samples.

### 3.3. Electrochemical performance of the CdO composite for energy storage

**Fig. 7:**
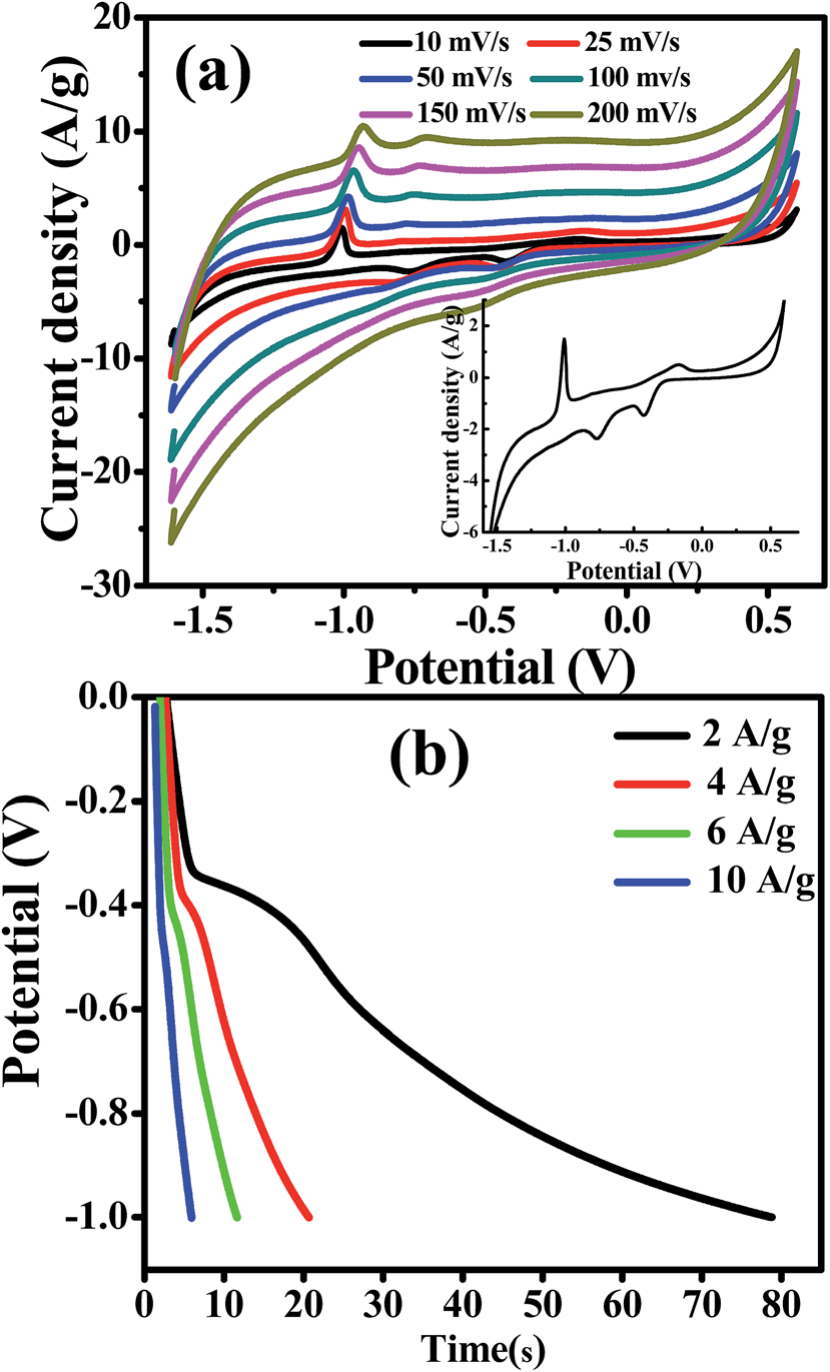
(a) CV curves of CdO nanocomposite in 1.0 M NaOH at different scan rates (inset; CV curve at 10 mV s^−1^), (b) galvanostatic charge/discharge curves of CdO composite in 1.0 M NaOH at different charge/discharge currents.

Cadmium hydroxide has been used as a promising electrode material for cadmium–nickel batteries for several decades due to its high energy density, long lifetime and high discharge rates. However, there are also a few literature reports considering Cd-based materials in supercapacitor applications, as shown in Table 2. Based on the large specific area of randomly stacked CdO/Cd(OH)_2_ nanoplatelets and their unique electrocatalytic capability through the presence of bi-nanocrystals of CdO/Cd(OH)_2_, it was expected that the CdO nanocomposite electrode would exhibit a high performance for energy storage supercapacitors. With a three-electrode configuration, the CdO nanocomposite-SPE as a working electrode, Pt as the counter electrode and Ag/AgCl as the reference electrode, then, the pseudocapacitive performance of CdO nanocomposite-SPE was improved. Cyclic voltammetry and galvanostatic charge–discharge measurements were carried out in 1.0 M NaOH with a three-electrode system at room temperature (Fig. 7). It can be seen that the CV curve exhibits several redox peaks, indicating that the capacitance is mainly governed by the pseudocapacitor behaviour based on the redox process, as following:^31^CdO + H_2_O + OH^−^ + e^−^ ↔ Cd(OH)_3_^−^Cd(OH)_3_^−^ + OH^−^ + e^−^ ↔ Cd(OH)_4_^2−^

**Table 2:** Comparative capacitance of cadmium-based electrodes

Materials	Electrolyte	Scan rate mV s^−1^	*C*_s_ (F g^−1^)	Current density (A g^−1^)	*C*_s_ (F g^−1^)	Ref.
CdO film on ITO	1 M Na_2_SO_4_	1.0	1.19	0.02	1.38	42
CdO NRs on platinum	0.1 M KCl	10.0	352	—	—	43
Cd(OH)_2_ nanowires on stainless steel	1 M NaOH	5.0	267	1.87	58.8	16
Cd-Doped Co/C in graphite shell	1 M KOH	5.0	255	—	—	44
Boron-doped CdO on stainless steel	1 M KOH	2.0	20.05	—	—	45
CdO/Cd(OH)_2_ on SPE	1 M NaOH	25.0	255	2.0	145.5	Present work
NiO NR-GCE	2 M NaOH	1.0	720	1.0	770	23
NiO-NPLs	2 M NaOH	1.0	1361	1.0	1200	24

The effect of scan rate on the CdO composite electrode was investigated in 0.1 M NaOH in the range from 0.01 to 0.15 V s^−1^. It is observed that the current density increases gradually, while the shape of the cyclic voltammetry curves remain unchanged with increasing scan rates. This result indicates the good pseudocapacitive behaviour of the CdO composite-SPE with a high rate capability. The specific capacitance of the CdO nanocomposite electrode was calculated from CV curves at different scan rates, as following:
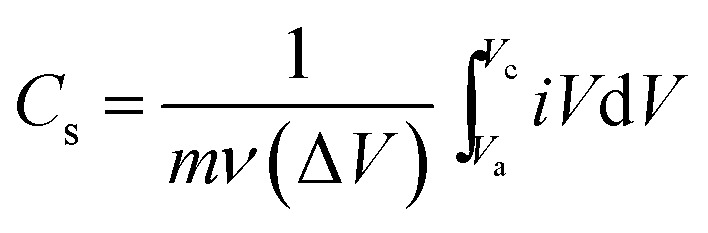
where *C*_s_ is the specific capacitance in F g^−1^, *m* is the mass of active material in grams, *ν* is the potential scan rate in V s^−1^, Δ*V* is the working potential window and *iV*d*V* is the integration area under the CV curve. The specific capacitance was calculated to be 253.28, 255.06, 229.65, 204.47, 191.48 F g^−1^ at potential scan rates of 0.01, 0.025, 0.05, 0.1, 0.15 V s^−1^, respectively. It was obvious that the specific capacitance was decreased with increasing the potential scan rate. Interestingly, the specific capacitance of the CdO nanocomposite-SPE was decreased about 13% with a ten-fold increase in the scan rate. This result indicates the high capability rate and applicability of the simply synthesized CdO nanocomposite compared to the previously reported supercapacitor based on CdO and/or Cd(OH)_2_ nanostructures, in which the specific capacitance decreases significantly with increasing the potential scan rate.^16,39–42^

**Fig. 8:**
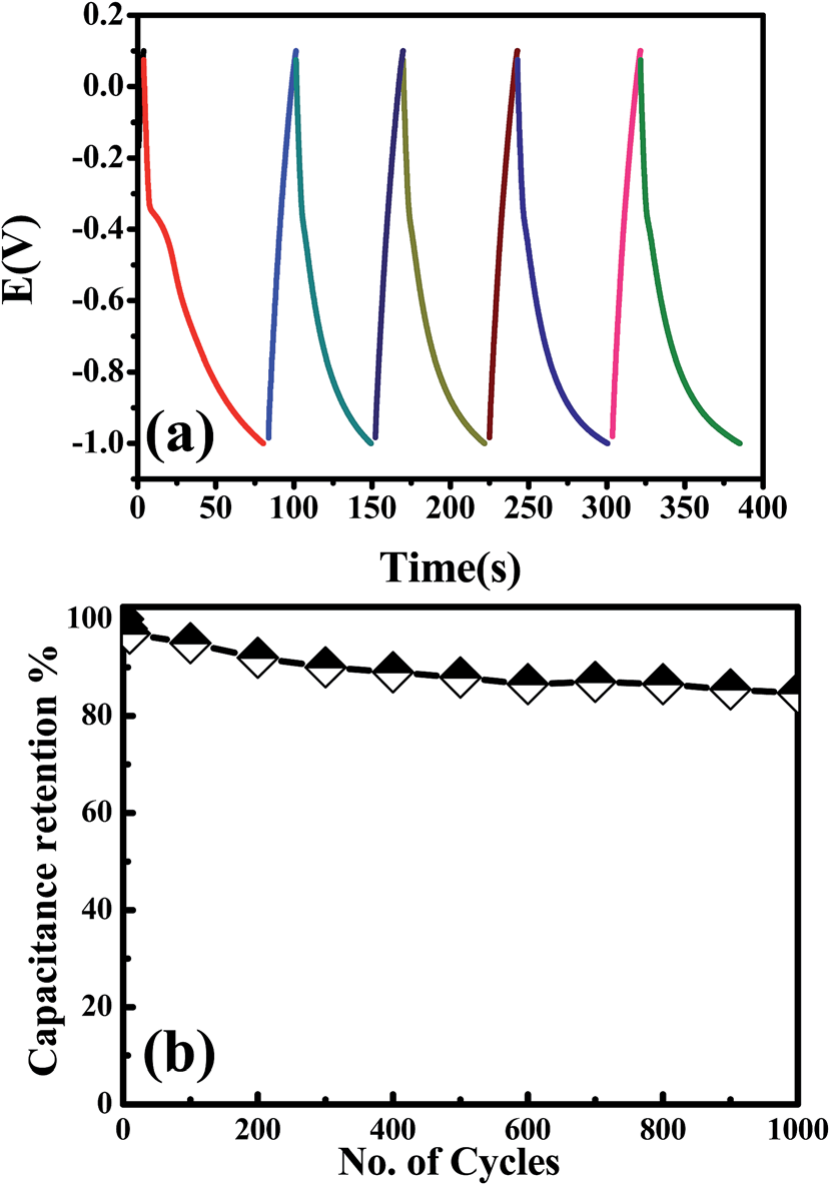
Applicability and cycling performance of CdO nanocomposite in 1.0 M NaOH at 2 A g^−1^.

To further estimate the specific capacitance (*C*_s_) and to comprehend the rate capability, galvanostatic charge–discharge measurements were performed within the potential window 0.0 to −1.0 V, at different current densities in 1.0 M NaOH, and the results are presented in Fig. 7b. The specific capacitance was measured by galvanostatic charge–discharge curves using the following equation:
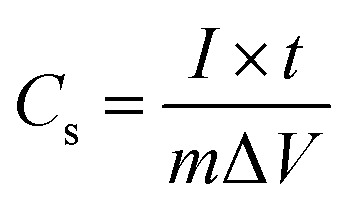
where *C*_s_ is the specific capacitance, *I* is the current response, *t* is the discharge time, *m* is the mass of electroactive material and Δ*V* is the working potential window. The specific capacitance was found to be 145.45 F g^−1^ at a current density of 2.0 A g^−1^. It was found that the specific capacitance, *C*_s_, was significantly enhanced more than two-times over the value reported for one-dimensional cadmium hydroxide nanowires^16^ and other reported Cd-based materials (Table 2). The specific capacitance was reduced with increasing the current density, which might due to the shorter diffusion path to reach the active sites of the cadmium oxide nanocomposite as well as the deficit of the faradic redox reaction at a higher current density. Noticeably, the specific capacitance remained as high as 87.02 F g^−1^, even when the discharge current density was increased to 8 A g^−1^. These results showed satisfactory long-term stability and a high specific capacitance due to their unique internal crystal structure (Fig. 8). As shown in Fig. 8, there is continuous galvanostatic charge–discharge cycling at a constant current density of 2 A g^−1^ over 1000 cycles. The CdO nanocomposite showed long-term stability compared with that previously reported (Table 2), although the specific capacitance lost about 7% in the first 200 cycles and then no further change was observed over the following 800 cycles. To the best of our knowledge, this interesting pseudocapacitive behaviour is rarely observed in Cd-based materials, which might be attributed to the presence of mixed nanocrystals of Cd(OH)_2_ in CdO nanoplatelets which offer a short diffusion path, fast electron/ion transfer and better stress/strain accommodation, which enhance its electrochemical performance.

## 4. Conclusions

The simple and large-scale production of hexagonal CdO/Cd(OH)_2_ nanocomposites with a platelet-like morphology was achieved through a partial thermal treatment of Cd(OH)_2_ platelets at 300 °C. Interestingly, the CdO/Cd(OH)_2_-SPEs showed high electrocatalytic activity towards nitrite oxidation over a wide concentration range with a lower micromolar detection limit achievable at pH 4 and formed a stable, high capability supercapacitor electrode in 0.1 M NaOH solution. The results suggested that the presence of Cd(OH)_2_ nanocrystals in the CdO nanoplatelets showed enhanced electrochemical performance with high stability, which might open new avenues in the fabrication of nanodevices based on electrochemistry with unique capabilities.

## Conflicts of interest

There are no conflicts to declare.

## Supplementary Material

RA-008-C7RA09457D-s001
