# Supplementary material for: Large-scale production of CdO/Cd(OH)2 nanocomposites for non-enzyme sensing and supercapacitor applications
Source: RSC Adv. 2018 Jan 3;8(2):921–30. doi: 10.1039/c7ra09457d (PMC9076989; doi:10.1039/c7ra09457d)
Supplement: RA-008-C7RA09457D-s001 [file RA-008-C7RA09457D-s001.pdf]

Electronic Supplementary Material (ESI) for RSC Advances.
This journal is © The Royal Society of Chemistry 2017

Electronic Supplementary Information (ESI)

Large scale production of CdO/Cd(OH)$_2$ nanocomposite for non-enzyme sensing and supercapacitor applications

Mohamed Khairy,[a*] Haytham A. Ayoub,[a] Craig E. Banks*[b]

S1:

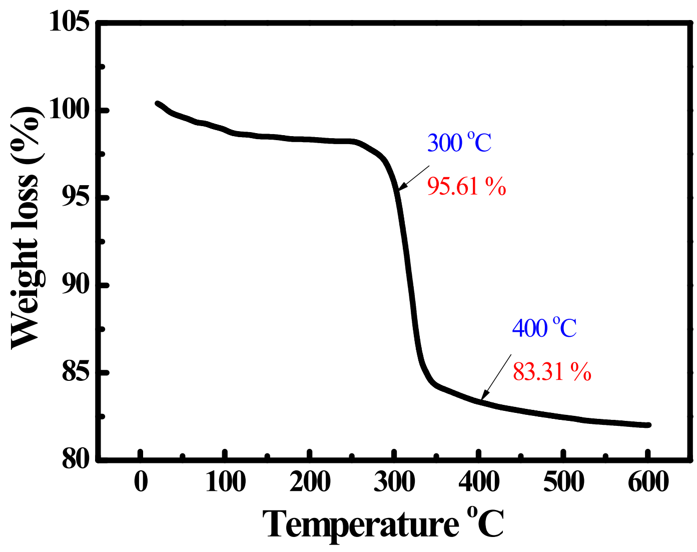

Figure S1. TGA curve of Cd(OH)$_2$ in nitrogen flow.

S2:

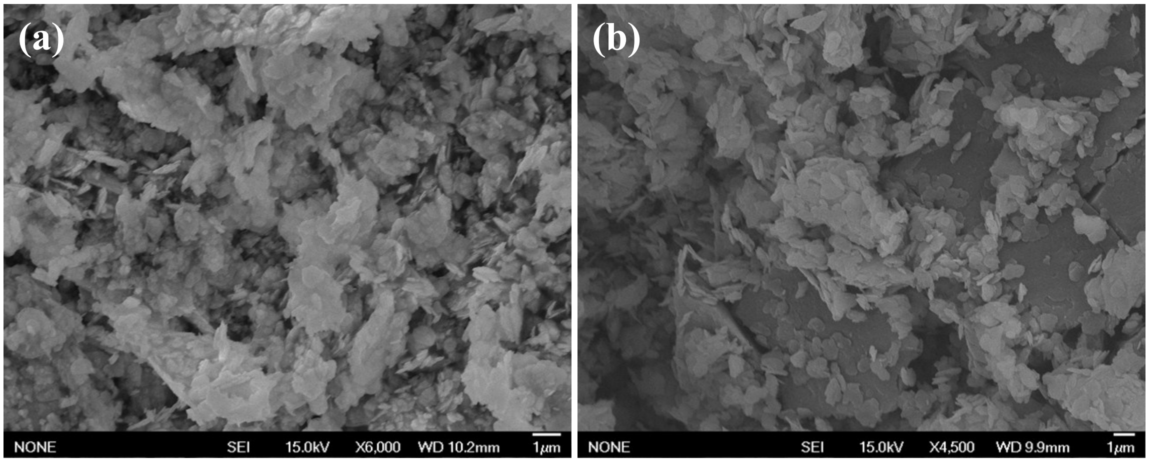

Figure S2. SEM images of Cd-based materials synthesized in (a) in presence of NaOH, pH = 9.5 and (b) in presence of NH_4OH, pH = 12 at 160 ^oC for 6 h.

S3

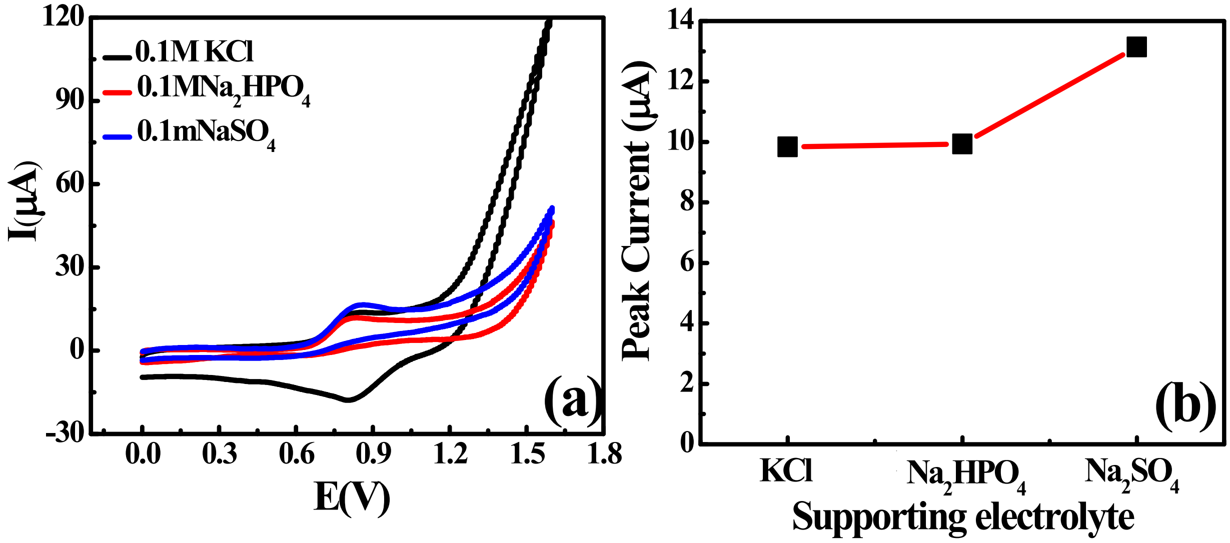

Figure S3. (a) CV curves of 0.5 mM of nitrite on CdO/Cd(OH)$_2$/ Au-SPE in 0.1 M different supporting electrolytes at scan rate 50 mV/s. (b) Analysis of peak current of nitrite oxidation in presence of different supporting electrolyte.

S4:

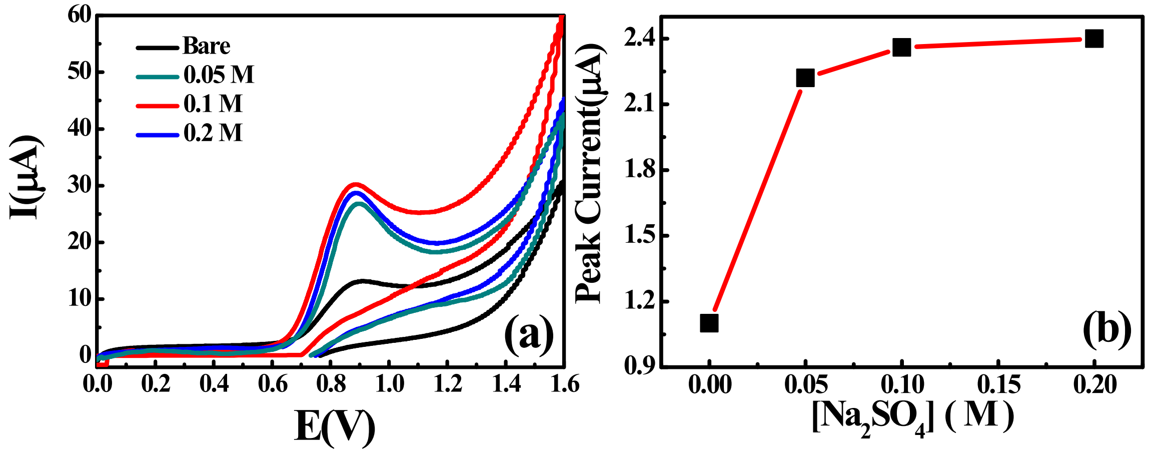

Figure S4. (a) CV curves of 1.0 $\times 10^{-3}$ M of nitrite ions at CdO/$Cd(OH)_2$/Au-SPEs in(0.0, 0.05, 0.1, 0.2 M) of Na_2SO_4 as supporting electrolyte, with scan rate 50 mV/s. (b) Analysis of peak current of nitrite oxidation in presence of different concentrations of Na_2SO_4.

S5:

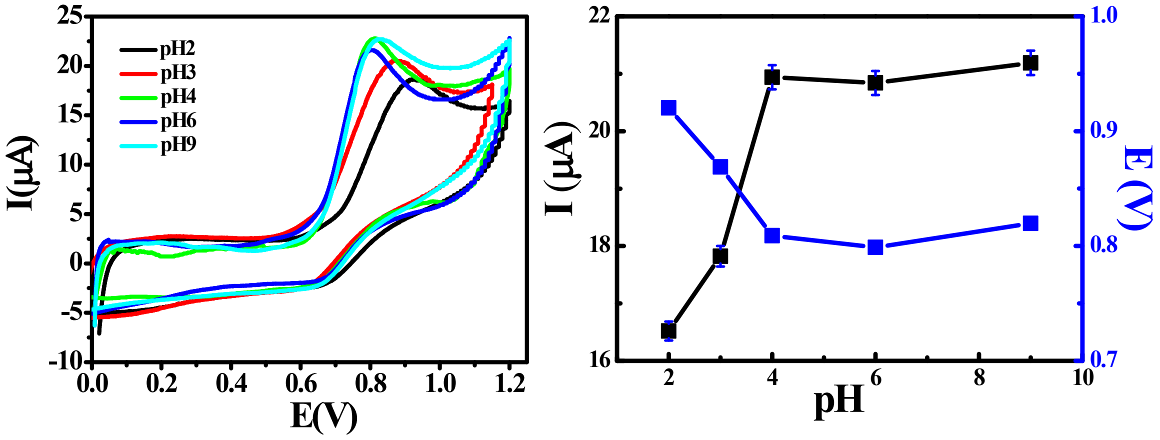

Figure S5. (a) CV curves of 1.0 mM nitrite on CdO/ $Cd(OH)_2$ /Au-SPE in different pHs at scan rate 50 mV/s, (b) Analysis of peak current and peak potential in different solution pHs .

S6

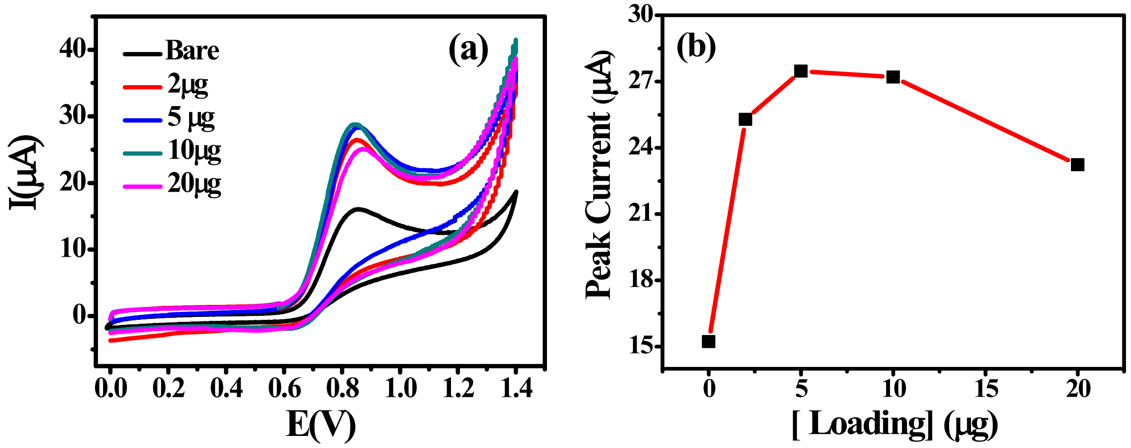

Figure S6. (a) CV curves of 1.0 $\times 10^{-3}$ M of nitrite ions at different mass loading of 0.0, 2, 5, 10, 20 µg $Cd(OH)_2$/ CdO /Au-SPEs in 0.1 M of Na_2SO_4 at scan rate 50 mV/s.(b) Analysis of peak current of nitrite oxidation and mass loading.

S7:

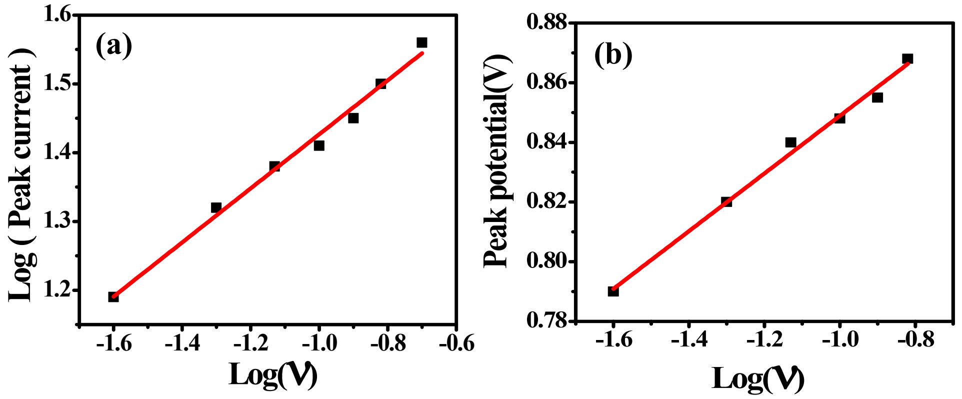

Figure S7. (a) Relation between log (current) vs. log scan rate, (b) potential dependence on log (scan rate); The voltammetric cell containing 1.0 mM sodium nitrite in 0.1 M of Na_2SO_4 (pH 4).

S8:

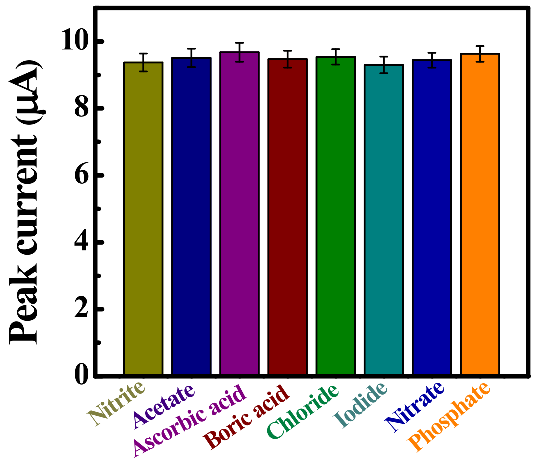

Figure S8. Voltammetric peak current analysis of 100 μM sodium nitrite in presence of 100 μM of sodium acetate, ascorbic acid, boric acid, potassium chloride, potassium iodide, sodium nitrate and sodium phosphate.

S9.

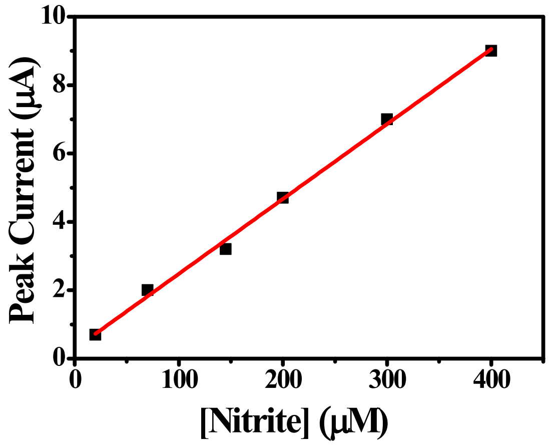

Figure S9. Standard additions of nitrite into a tap water sample using CdO composite modified screen-printed electrode. The sample was simply acidified to pH 4 with a small amount of 0.1 M sulfuric acid.
